# Facilitating reproducible project management and manuscript development in team science: The projects R package

**DOI:** 10.1371/journal.pone.0212390

**Published:** 2019-07-29

**Authors:** Nikolas I. Krieger, Adam T. Perzynski, Jarrod E. Dalton

**Affiliations:** 1 Department of Quantitative Health Sciences, Lerner Research Institute, Cleveland Clinic, Cleveland, Ohio, United States of America; 2 Center for Healthcare Research and Policy, Case Western Reserve University at MetroHealth, Cleveland, Ohio, United States of America; 3 Cleveland Clinic Lerner College of Medicine, Case Western Reserve University, Cleveland, Ohio, United States of America; University of Cyprus, CYPRUS

## Abstract

The contemporary scientific community places a growing emphasis on the reproducibility of research. The projects R package is a free, open-source package created in the interest of facilitating reproducible research workflows. It adds to existing software tools for reproducible research and introduces several practical features that are helpful for scientists and their collaborative research teams. For each individual project, it supplies a framework for storing raw and cleaned study data sets, and it provides script templates for protocol creation, data cleaning, data analysis and manuscript development. Internal databases of project and author information are generated and displayed, and manuscript title pages containing author lists and their affiliations are automatically generated from the internal database. File management tools allow teams to organize multiple projects. When used on a shared file system, multiple researchers can harmoniously contribute to the same project in a less punctuated manner, reducing the frequency of misunderstandings and the need for status updates.

## Introduction

The past few years have yielded much discussion and controversy in scientific circles surrounding reproducibility and replication of findings. While the terms “reproducibility” and “replicability” have sometimes been used interchangeably [1], it is important to address the nuances that make these concepts distinct. Reproducibility can be defined as the extent to which readers can emulate the researchers’ methods and workflow, while replicability is more commonly viewed as the extent to which study results generalize to external samples.

As its name implies, the *replication crisis* most directly concerns the concept of replicability of findings, but it also implicates the related concept of reproducibility, which is a prerequisite for conducting replication studies. While there may be many reasons why a given hypothesis or phenomenon fails to replicate in external samples, we believe that the lack of a consistent analytic workflow should not be among those reasons. Unfortunately, several meta-scientific studies demonstrate that numerous published manuscripts lack navigable workflows or are not reproducible [2–5]. Actively maintaining and archiving the workflow that engenders a manuscript is crucial to its proper evaluation. There exist today widely available tools that aid with reproducible research, such as R and other statistical programming languages, that allow for precise documentation of some of the most detail-oriented portions of a project workflow. [6] Researchers can distribute code scripts alongside results in order to communicate the integrity of data processing and analysis. Unfortunately, statistical programming tools and code only contribute to research reproducibility insofar as individual statistical programmers are able (i) to use these tools effectively and (ii) to integrate their own use of these tools with their collaborators’ work—which may not be oriented towards reproducibility.

In this paper, we introduce the projects R package, a set of tools that supports an efficient project management workflow for statisticians and data scientists who perform reproducible research within team science environments. Deliberately oriented towards academic manuscript development, projects is one element of a growing ecosystem of reproducibility-facilitating software. [7,8] As such, it is built upon some existing tools in that ecosystem. Being a fundamentally interactive package, projects was designed in and works in concert with RStudio, an interactive R integrated development environment. [9] It also promotes and employs R Markdown (.Rmd), a file structure accommodating text, code and code output. [10] Fully integrated with RStudio, R Markdown allows users to interleave statistical programming code with formatted annotations, enabling users to write highly technical documents. R Markdown files can then be rendered as submission-ready manuscript files (in .html, .pdf or .docx format) via the knitr R package [11].

The projects package extends upon these capabilities by introducing a dedicated manuscript-oriented workflow, enabling researchers in any field to build and archive reproducible analytic workflows. It offers a unified approach to data management, analysis, and reporting that is common to almost all manuscript-oriented research. The package accomplishes this in part by automatically generating protocol and analysis templates in the R Markdown file format. The pre-loaded protocol templates are taken from the Gmisc R package and are based on widely accepted reporting guidelines, such as the Consolidated Standards of Reporting Trials (CONSORT) statement and the Strengthening the Reporting of Observational Studies in Epidemiology (STROBE) recommendations [12–14]. The projects package not only encourages individual researchers to utilize reproducibility facilitation tools—it also encourages the use of R Markdown and RStudio by collaborative research teams by providing infrastructure for team science: when used on a shared file system (e.g., a server), multiple researchers can work on the same project and keep track of its progress without having to request updates. This removes significant logistical and motivational barriers that otherwise accompany the passing of manuscript drafts back and forth; projects makes existing reproducibility facilitation tools more accessible to both individuals and collaborative groups.

We present below the conceptual framework of a manuscript-oriented workflow, followed by a list of the primary features of the projects R package, which delineates how the package works to address reproducibility. We continue with a description of the package itself, detailing its approach to the conceptual framework and including links to a mock project to illustrate its use. Lastly, we discuss the unique role that projects can have in the ecosystem of reproducibility-facilitating software, comparing and contrasting the package with other such R packages.

## Conceptual framework: Reproducible research workflows

Although researchers of different disciplines may conduct their daily research work in many nuanced ways, there are aspects of the project workflow that are common to most investigations. First, studies are conceptualized and designed according to a protocol that details the research questions and planned analyses. Data are collected, manipulated (or “tidied”) in order to make data analysis possible. The results of the analyses are compiled into a report, and ultimately an academic manuscript is drafted and submitted for wider distribution.

When navigating this workflow, researchers strive for reproducibility wherever possible but especially during the intermediate, data-focused phases of the workflow. While primary data sources for research projects can be complex, dynamic and diverse, a reproducible analytic workflow should if possible incorporate a “frozen” dataset that reflects a given set of queries or data collection forms at a specific point in time. A frozen dataset represents the study data’s earliest state of simultaneous digitization and consolidation and is almost invariably a digital file or set of files that standard data analysis software can process (e.g., a comma-separated values (.csv) file; a series of .csv files; a compressed-format dataset in the researcher’s analytic programming language of choice).

**Fig 1:**
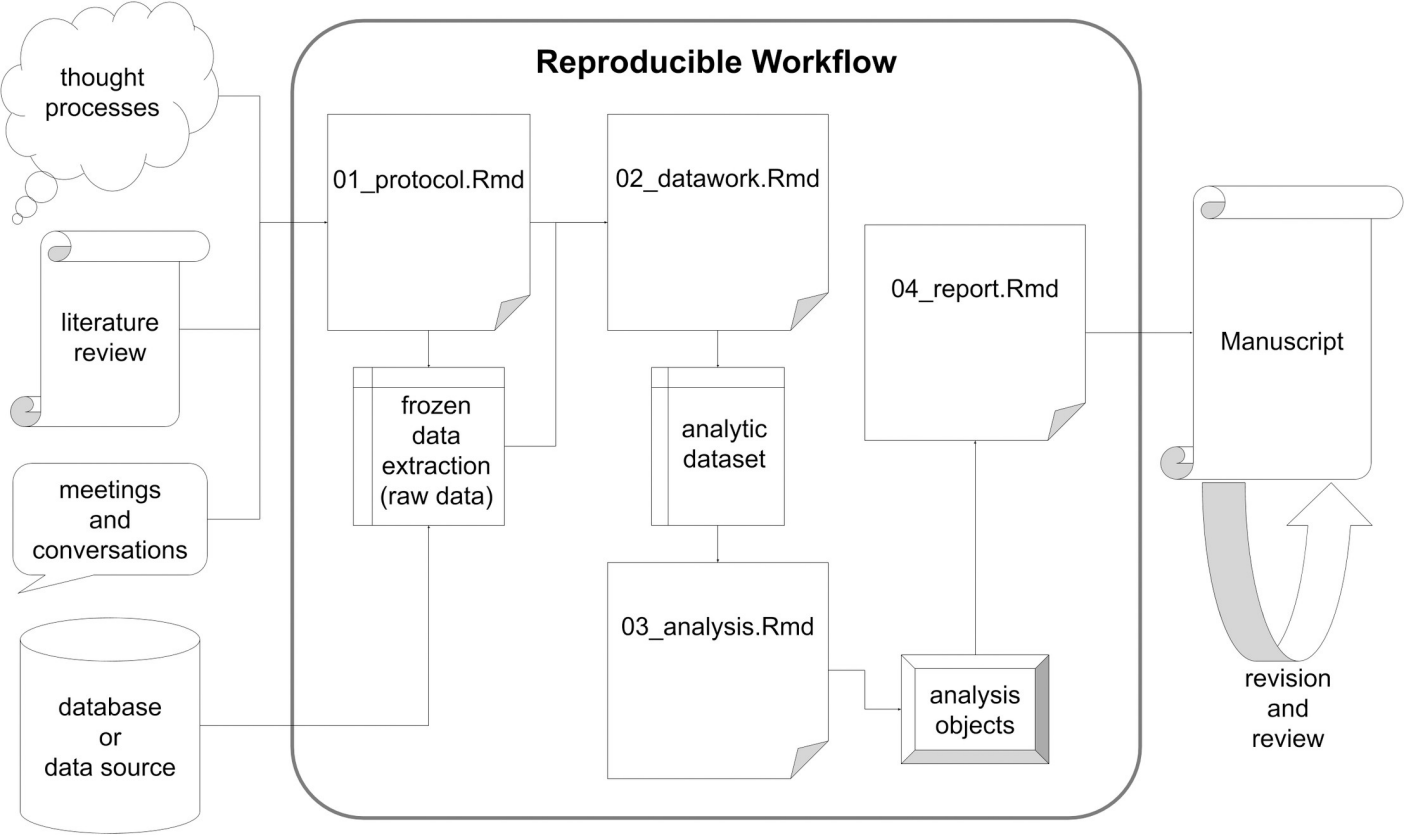
The entire workflow of manuscript creation with the reproducible portions encircled.

The structure of this data set can then be used as a basis for replicability studies, in that the data manipulation and analytic programming steps can be exported to other settings (assuming the data structures are consistent—see Fig 1). Thanks to modern data analysis via statistical programming languages, a reader should be able to exactly reproduce all data-derived results from the frozen dataset alone. Under the reproducible research framework, other researchers with access to the original data and code scripts can scrutinize every component of the analysis, beginning with data cleaning operations performed on their local version(s) of the frozen dataset.

The middle stage of the assumed study workflow can typically be performed with near perfect reproducibility, but the beginning and ending stages may not. Researchers cannot document every thought process, literature probe and informal conversation that contributes to the development of the initial study protocol, but they should strive to document it as meticulously as possible. Databases tend to be dynamic such that a given analytic dataset is merely a snapshot in time. As for the final stages of project development, journals require that manuscripts adhere to specific and unique stylistic guidelines and that they be digitally submitted with file types that are not independently conducive to reproducibility (e.g., .pdf). The ultimate goal of addressing a *replication* crisis beseeches researchers to pursue *reproducibility* and therefore to adopt consciously a strategy that reliably effectuates reproducibility; the projects package offers one such strategy.

### The projects R package

The projects package is open source and maintained on the Comprehensive R Archive Network. The package can be installed from the R console using the following command:

install.packages("projects")

Development versions of the package can be found on the projects GitHub page at https://github.com/NikKrieger/projects and installed using:

remotes::install_github("NikKrieger/projects")

The primary features of the projects R package are the following:

- A manuscript-oriented reproducible research workflow with dedicated document templates for each stage of the manuscript development process;
- Automated project file and folder generation in an organized structure;
- Customizable templates that decrease the administrative overhead of project structuring, freeing users to focus on actual research;
- Organization and management functionality, including the ability to create, edit, group, archive and delete projects;
- Integration with the unique benefits of R Markdown that expedite the production of academic manuscripts;
- Relational database containing details of projects, project coauthors and their affiliations, centralizing project status information and automating the creation of title pages;
- Utilization of RStudio for an interactive interface;
- Saving of R session information with the running of a single function;
- Exporting of entire or partial contents of project folders for sharing.

### The */projects/* folder file structure

To start, the user runs the function setup_projects() in order to create the main */projects/* folder in a user-specified location. Henceforth, the user can run the flagship function of the package, new_project(), in order to create a new project folder within the main */projects/* folder. Each new project folder automatically includes aptly named subfolders and templates in order to guide the user along a reproducible project workflow. See, for example, the file structure for the newly created project 12:


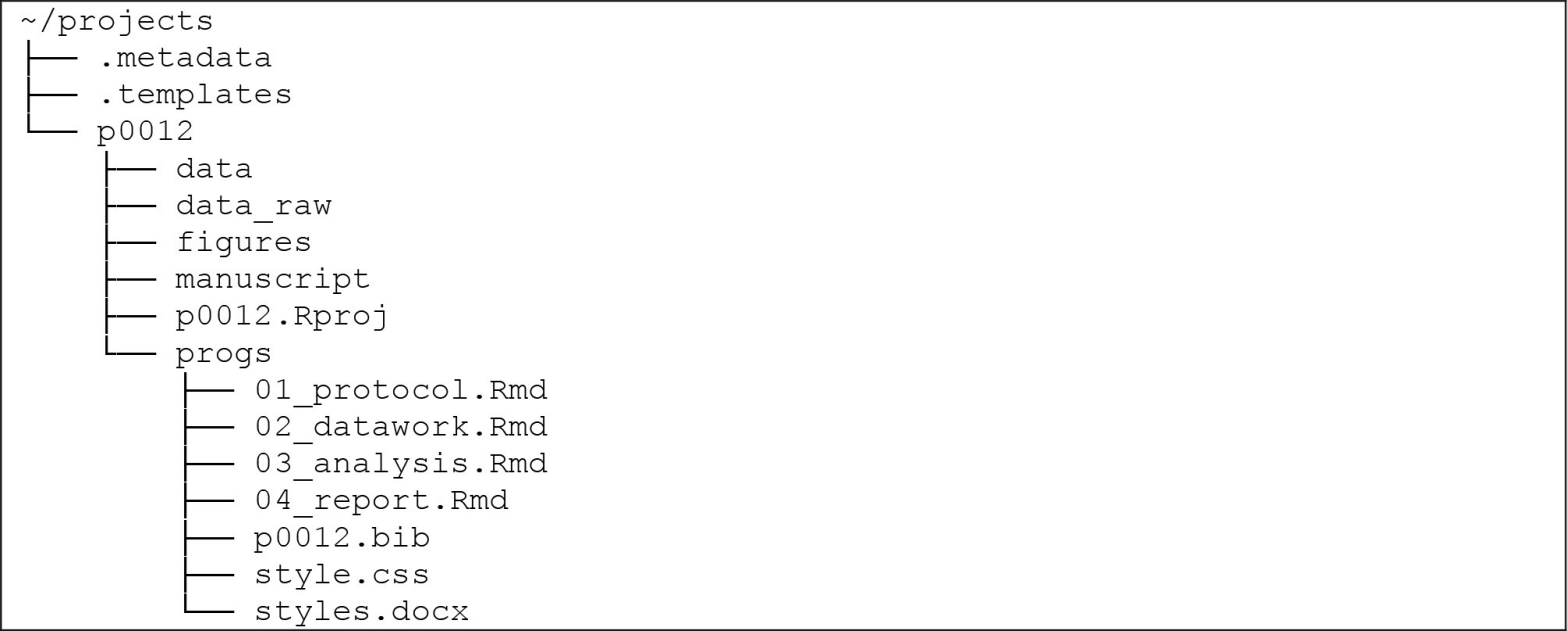


The subfolders serve to organize the project, providing intuitive locations to place files so that a user’s collaborators—and indeed the same user at a later date—may easily locate them. The .Rmd project template files in the */progs/* (short for “programs”) folder form a chain that when followed produces a self-contained, reproducible workflow. Most of the user’s effort will be focused on these files: all other features of projects serve to assist with the composition and organization of these files. Project folders can also be exported and shared using the export_project() function.

### Project workflow

Manuscript-oriented projects all follow a general workflow. First, studies are conceptualized and designed in a protocol document, which details the research questions and planned analyses. Data are then collected, manipulated (or “tidied”) in order to make data analysis possible. The results of the analyses are compiled into a report, and ultimately an academic manuscript is drafted and submitted for wider distribution. For each project, the projects package generates series of .Rmd files that guide researchers along this workflow. One important task involved with this .Rmd generation is the writing of YAML (YAML Ain’t Markup Language) headers at the top of each file. A YAML header is comprised of meta-data pertaining to the .Rmd, such as its title, authorship, and the names of other files containing citations or styling specifications. [10] The default .Rmd templates contains code for the importing and exporting of data and other files, and in doing so they guiding users towards the use of the here() function in the here package for streamlined file management. [15] Furthermore, at the end of each of these templates is a call to the projects function save_session_info(), which saves the R session information to a dated file, thereby documenting the crucial information necessary to reproduce the script. A mock project displaying the contents of these .Rmds as well as their use in the projects workflow is available at https://github.com/NikKrieger/projects/tree/master/demonstration.

#### 01_protocol.Rmd

During the conceptualization and design stage of a project, researchers may write a protocol, in which they explain the research questions they plan on answering and the manner and methods in which they plan to answer those questions. As mentioned above, the package contains two different protocol templates, one each according to the STROBE (the package default) and CONSORT guidelines. Since a protocol is normally a published document, the projects package writes authorship information into the YAML header of the 01_protocol.Rmd file in newly created projects. The user also has the option to make this file BibTeX-ready by setting use_bib = TRUE in the new_project() function: doing so causes projects to write a blank .bib file into the */progs/* folder and to include a bibliography line in the YAML that references this .bib file.

#### 02_datawork.Rmd

This .Rmd file is intended for the importing, cleaning, and exporting of data. Once users have placed raw data in the project’s */data_raw/* folder, they can use 02_datawork.Rmd to import it into R and to clean it. When the data are prepared for analysis, users can write a save() function to export all analysis-ready objects into the project’s */data/* folder. Hence, these prepared data are available to the next .Rmd file for analysis.

#### 03_analysis.Rmd

Similar to 02_datawork.Rmd, this file is intended for the importing, processing, and exporting of objects. In this case, clean data are imported from the */data/* folder and analyzed using desired statistical methods. Resulting tables and figures are exported back into the */data/* folder to be gathered by the next .Rmd for reporting.

#### 04_report.Rmd

This file is the last .Rmd file in this sequence. It imports analysis objects created in 03_analysis.Rmd and integrates them into the manuscript text. Like the 01_protocol.Rmd templates, since its result is a publishable manuscript, the default 04_report.Rmd also contains the project authorship and an optional bibliography line in its YAML header upon creation. Unlike the protocol templates, it is brief overall, as there is wide variation in journal submission requirements.

#### Customization

Users may desire to modify or replace the default templates described above to serve their specific research needs. Upon creation, the main */projects/* folder contains a subfolder called */*.*templates/* that contains the default templates for the aforementioned .Rmd files as well as a few other files:


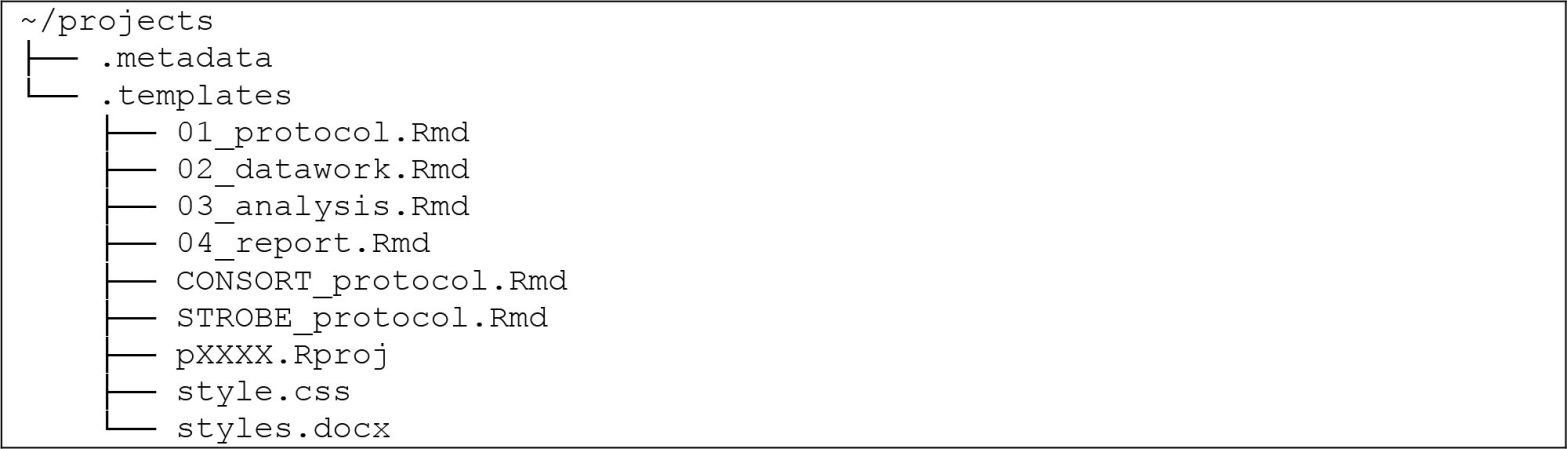


A selection of these templates is copied into each newly created project folder. The function new_project() selects the templates by name according to its arguments. The templates with the names listed above are chosen by default, so the user can edit these default templates within */*.*templates/* to meet their research and reproducibility needs. If users require different templates for different situations, they can add different templates to the */*.*templates/* folder and choose the desired template on each execution of new_project().

In addition to the .Rmd project file templates, there is a template for the .Rproj file that RStudio uses in its project-oriented environment. There are also templates for optional style customization files, style.css and styles.docx, which rmarkdown can utilize to style rendered .html and .docx files, respectively [10]. Users can create and edit these style customization files in order to obtain the desired font, paragraph spacing, text size, and so forth.

### Project management

Acknowledging that researchers create, move, copy, delete and archive files on a daily basis, one of the goals of the projects package is to provide a comprehensive set of tools for managing project files in a way that is self-contained in R and independent of the underlying operating system. It offers interactive functions to perform all these actions in an organized manner with an automated and predictable file structure. In fact, users are advised not to move the */projects/* folder nor individual project folders (e.g., */projects/p0012/*) with their operating system, so that the package does not lose track of these files. Multi-user application of projects requires a server or an otherwise shared directory where multiple users can access the */projects/* folder. Since projects created via projects are contained within a single folder that contains a .Rproj file, users interested in version control can utilize version control capabilities integrated with RStudio (e.g., Git, Subversion) as they would with any other RStudio-housed project.

### Project meta-data and title pages

In addition to the template-oriented arguments mentioned above, new_project() contains several other arguments that allow the user to populate the project meta-data. Alongside project titles and author lists, project meta-data include development-oriented information like project stage and deadline date. These meta-data are stored in */projects/*.*metadata/* and are viewable using the projects() function. Members of a team that uses projects can run this function at any time to check its status, and as it develops into a manuscript these collaborators can update the meta-data using edit_project().

Concerning project authorship in particular, since projects and authors have a many-to-many relationship, project authorship is stored as a relational database within */*.*metadata/*. The functions new_author(), authors() and edit_author() correspond to the similarly named project-related functions mentioned above. Furthermore, users may add, recall and update authors’ affiliation information (i.e., where they work and the institutions to whom they belong) using new_affiliation(), affiliations() and edit_affiliation(). One of the main benefits to logging all these data is that it enables the package to generate the code and text necessary for rmarkdown and knitr to generate properly formatted title pages for finalized protocols and manuscripts, lessening the burden of one of the most mundane tasks that manuscript writing involves.

## Discussion

The projects package provides a novel set of tools for reproducible team science workflows. It directs researchers to a consistent workflow based on a sequence of structured R Markdown files, which fosters reproducibility and encourages compliance with reporting guidelines. While the package allows for customization, default settings encourage use of the here package to make file paths generalizable; thus, code can successfully access data and other files within the projects folder, no matter where it is located within the file structure of the computer or server. The meta-database feature facilitates efficiency in both project management and manuscript writing: users can view and record project statuses and details as well as team members’ affiliations and contact information. For manuscripts, title pages are automatically generated from the meta-database.

Other workflow-related R packages exist, such as workflowr, which offers collaborative project file management on GitHub. [7] The projects package, however, is unique in its focus on manuscript development, being created by and for researchers who submit their research to academic journals. Furthermore, while GitHub is an excellent tool for facilitating reproducibility and replicability, some academic research settings disallow its use—even the use of its “private” data-hosting services. Research teams who regularly utilize firewalled data may still use projects, which may operate on the file system of a secure network.

ProjectTemplate is another workflow-related R package that organizes project files. [8] In terms of organization, it goes further by managing the R packages that the user will use in their data analysis; it also partially automates the attaching of these packages, the loading of project data, the cleaning of the data, and unit testing of data-related tasks. Users of projects run its functions at the beginning and end of sessions dedicated towards a project, while users of ProjectTemplate run its functions during these sessions. Therefore, whereas ProjectTemplate is focused on directly assisting user with the hands-on tasks of data cleaning and analysis, the projects package helps the user to weave the fruits of their data work and analyses into an academic manuscript. It is plausible that researchers may find it beneficial to use both packages within the same project.

The main limitation of the projects package lies in its setting: it is confined to the R statistical programming language, which not all researchers know and use. Prospective users of the package who do not know R must spend time learning how to use it, and this drawback is compounded as the size of the transitioning research team increases. Researchers who do know R may need to spend time gaining proficiency with R Markdown, knitr and other projects package dependencies. In future work, we will explore ways to integrate the functionality of the projects package with other statistical programming languages (e.g., Python and SAS) and with cloud-based collaborative tools. Additionally, since project teams will almost always include experts who do not use RStudio, the collaborative manuscript editing process usually finishes in a word processing environment (e.g., Microsoft Word) that only supports total reproducibility with extraordinary effort. In light of these realities, during manuscript creation researchers must do their best, keeping the process in reproducible environments for as long as possible and otherwise documenting significant changes and alterations.

In spite of the inevitable learning curve that is present when adapting to a new programming language, the projects package is intuitive to use among regular users of R and R Markdown. We believe that the projects package may be useful for teams that manage multiple collaborative research projects in various stages of development. It has the potential to improve both the quality and efficiency of individual and team researchers while also rendering the task of maintaining reproducibility less cumbersome. The open-source nature of the R environment ensures that the projects package will only improve with time and use, as the scientific community continues to embrace the tools essential for maintaining a reproducible workflow.
